# Evaluation of the cost-effectiveness of dexrazoxane for the prevention of anthracycline-related cardiotoxicity in children with sarcoma and haematologic malignancies: a European perspective

**DOI:** 10.1186/s12962-020-0205-4

**Published:** 2020-02-10

**Authors:** Sarah Dewilde, Kevin Carroll, Emilia Nivelle, James Sawyer

**Affiliations:** 1 Services in Health Economics, Brussels, Belgium; 2 KJC Statistics, Wilmslow, UK; 3 Prism Ideas Ltd, Morston House, Beam Heath Way, Nantwich, CW5 6GD UK

**Keywords:** Cost-effectiveness, Dexrazoxane, Prevention, Anthracycline, Cardiotoxicity, Sarcoma, Haematologic malignancy

## Abstract

**Background:**

Anthracycline-treated childhood cancer survivors are at higher risk of cardiotoxicity, especially with cumulative doses received above 250 mg/m^2^. Dexrazoxane is the only option recommended for cardiotoxicity prevention in high-risk patients supported by randomised trials but its cost-effectiveness in paediatric cancer patients has not been established.

**Methods:**

A cost-effectiveness model applicable to different national healthcare system perspectives, which simulates 10,000 patients with either sarcoma or haematologic malignancies, based upon baseline characteristics including gender, age at diagnosis, cumulative anthracycline dose and exposure to chest irradiation. Risk equations for developing congestive heart failure and death from recurrence of the original cancer, secondary malignant neoplasms, cardiac death, pulmonary death, and death from other causes were derived from published literature. These are applied to the individual simulated patients and time until development of these events was determined. The treatment effect of dexrazoxane on the risk of CHF or death was based upon a meta-analysis of randomised and non-randomised dexrazoxane studies in each tumour type. The model includes country specific data for drug and administration costs, all aspects of heart failure diagnosis and management, and death due to different causes for each of the five countries considered; France, Germany, the UK, Italy, and Spain.

**Results:**

Dexrazoxane treatment resulted in a mean QALY benefit across the five countries ranging from 0.530 to 0.683 per dexrazoxane-treated patient. Dexrazoxane was cost-effective for paediatric patients receiving anthracycline treatment for sarcoma and for haematologic malignancies, irrespective of the cumulative anthracycline dose received. The Incremental Cost Effectiveness Ratio (ICER) was favourable in all countries irrespective of anthracycline dose for both sarcoma and haematological malignancies (range: dominant to €2196). Individual ICER varied considerably according to country with dominance demonstrated for dexrazoxane in Spain and Italy and ratios approximately double the European average in the UK and Germany.

**Conclusions:**

Dexrazoxane is a highly cost-effective therapy for the prevention of anthracycline cardiotoxicity in paediatric patients with sarcoma or haematological malignancies in Europe, irrespective of the healthcare system in which they receive treatment. These benefits persist when patients who receive doses of anthracycline > 250 mg/m^2^ are included in the model.

## Background

Anti-cancer treatments, such as anthracycline therapy and radiation therapy, are used widely in the treatment of childhood cancer, particularly in a wide spectrum of solid organ and haematologic malignancies including leukaemia, lymphoma and sarcomas. Approximately 50–60% of childhood cancer survivors have been treated with an anthracycline regimen [1]. These treatments have several late adverse effects, with cardiotoxicity being one of the most widely recognised [2, 3].

Cardiotoxicity caused by anthracycline therapy manifests along a continuum from asymptomatic left ventricular dysfunction (ALVD) (a surrogate measure of anthracycline-induced cardiotoxicity) to congestive heart failure (CHF) [4]. Cumulative anthracycline dose is an independent risk factor for CHF, with higher cumulative doses, especially > 250 mg/m^2^, leading to higher risk of cardiotoxicity [5]. However, even at lower doses, subclinical cardiotoxicity may be observed, and lower doses may compromise efficacy [6]. Children with sarcomas are frequently treated with some of the highest cumulative anthracycline doses, making them particularly vulnerable to cardiac injury [7, 8].

In developed countries, total expenditure on CHF ranges between 1 and 2% of the total healthcare budget, with medical costs increasing with extent of left ventricular systolic dysfunction and the severity of the disease [9]. Direct medical costs include those associated with initial investigations and with treatment.

Intravenous dexrazoxane is currently the only agent recommended for cardiotoxicity prevention in high-risk patients [10]. Until recently, dexrazoxane use was restricted to adults with advanced or metastatic breast cancer who had already received at least 300 mg/m^2^ of doxorubicin or equivalent cumulative dose of another anthracycline. In its 2017 update to the European SmPC, the EMA noted that there is no evidence to support previous concerns about an increased incidence of second malignant neoplasm (SMN) in patients who have received dexrazoxane and modified the contraindication, limiting its administration to paediatric patients planned to receive > 300 mg/m^2^ of doxorubicin or equivalent cumulative dose of another anthracycline. Follow up results among 1022 patients from the Children’s Oncology Group (COG) AAML0531 study strongly indicate that the occurrence of left ventricular dysfunction whilst receiving cancer treatment was associated with poorer oncologic treatment outcomes [11].

The cost-effectiveness of dexrazoxane in paediatric cancer survivors is yet to be established. Here we present a cost-effectiveness model, based on European-wide healthcare data and costs, that aims to assess the budget impact and cost-effectiveness of dexrazoxane in children with sarcoma or haematological malignancies.

## Materials and methods

### Model design

A cost-effectiveness model applicable to different national healthcare system perspectives across Europe was developed. This model was populated with data for France, Spain, Italy, Germany, and the United Kingdom (UK).

**Fig. 1:**
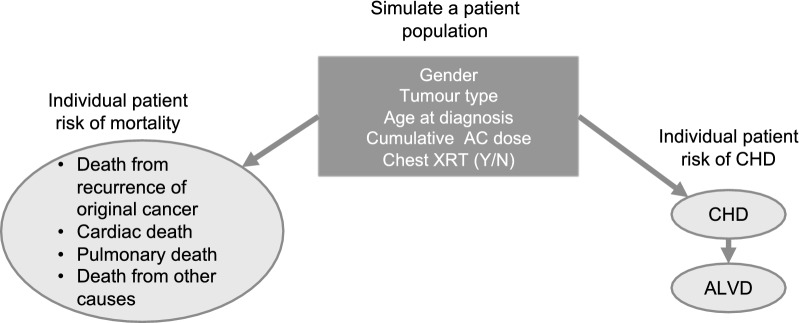
Model schematic. AC, anthracycline; ALVD, asymptomatic left ventricular disease; CHF, congestive heart failure; XRT, chest irradiation

The model simulated children and adolescent patients with either sarcomas or various haematologic malignancies (including acute myeloid leukaemia, acute lymphoblastic leukaemia, Hodgkin lymphoma and non-Hodgkin lymphoma) who survived for 5 years after diagnosis and who received treatment with chemotherapy regimens containing an anthracycline, such as doxorubicin or epirubicin. Each patient had a set of baseline characteristics including sex, tumour type, age at cancer diagnosis, cumulative anthracycline dose, and exposure to chest irradiation (Fig. 1). The baseline parameters were based on data from Cancer Research UK [12].

The same national cohorts entered two treatment arms in the model: patients having received dexrazoxane versus those who had not. Three different dose thresholds for anthracycline exposure were employed in the model to allow for different treatment paradigms and clinical eventualities (exposure to any dose of anthracycline, > 250 mg/m^2^, and > 100 mg/m^2^). These thresholds were also chosen in order to determine the cost-effectiveness of dexrazoxane when initiated at the start of anthracycline therapy or after prolonged treatment with a higher anthracycline dose.

A targeted literature review was conducted in May 2018 to identify clinical information and economic evidence describing healthcare resource use, costs, and quality of life (QoL) utility values associated with anthracycline administration and the management of cardiac failure in each of the five countries addressed by the model. This literature review also sought to identify risk equations to link surrogate outcomes to patient outcomes, such as heart failure and other cardiac events. Finally, the review identified all published clinical trials evaluating the addition of dexrazoxane to anthracycline therapy in the treatment of childhood cancer (see Additional file 1). To maximise the volume of outcomes data that could be included in the model, the search strategy included studies where dexrazoxane was administered at 10:1 to doxorubicin (the current recommended ratio) or at 20:1 to doxorubicin (the historical regimen).

Several patient subgroups were considered in the model based on additional parameters known to affect lifetime risk of cardiotoxicity, for example age at treatment initiation, gender, or chest irradiation.

The model was programmed with a base case setting using the most robust, conservative data plus other programmed scenario analyses, such as one of three anthracycline administration dose ranges (any anthracycline dose; > 250 mg/m^2^; and > 100 mg/m^2^), to allow the user to select alternative data sources for the key clinical events in the model.

### Clinical inputs

Known risk equations from the published literature were applied to the individual simulated patients. Baseline risk of developing CHF was based upon risk equations and cumulative incidence graphs presented in the standard risk model developed by Chow et al. [13], as it best matched the available input variables from dexrazoxane studies, in which data from the US Childhood Cancer Survivor Study (CCSS) was used to predict heart failure in survivors of childhood cancer where clinical dose information was known [3, 14]. For the purpose of this base case analysis, the chance of an individual patient receiving chest irradiation was set at 18%, based on the incidence of chest irradiation reported in a large retrospective study of dexrazoxane in children with anthracycline-induced cardiotoxicity [15]. An alternative risk model for CHF was developed for the sensitivity analysis, based on the results of a retrospective analysis of cardiac outcomes in adult survivors of the CCSS cohort [16]. This model included the impact of anthracycline dose, age at diagnosis, and gender on the risk of CHF, but, unlike that of Chow, only included two anthracycline dose categories and did not account for chest radiation. The risk of developing ALVD was estimated at three times greater than the risk of developing CHF [17–19], calculated according to age. Polynomial extrapolation of the cumulative CHF incidence was used for the base case, whereas linear extrapolation was used for the sensitivity analysis (Table 1).

**Table 1:** Data sources for base case and sensitivity analyses

Inputs	Base case	Sensitivity analysis
Risk of developing CHF	Based on risk scores and the standard model risk equations developed by Chow et al. [13]	Based on the Hazard ratio for CHF in a cohort of adult survivors of childhood and adolescent cancer according to anthracycline dose [16]
Extrapolation of cumulative CHF incidence	Polynomial	Linear
Risk of dying from non-CHF causes	Based on cause-specific cumulative mortality curves developed by Mertens et al. [20]	Based on life tables combined with SMR [21]
Treatment effect calculations	Based on meta-analysis of data from non-randomized and randomized studies using M–H approach	Based on Bayesian meta-analysis of data from non-randomized and randomized studies
Utility values	As presented in Wong et al. [22]	Based on New York Heart Association classes I and III, [0.855 (0.845; 0.846) and 0.673 (0.665; 0.690)] representing heath states ALVD and CHF respectively [23]

*ALVD* asymptomatic left ventricular dysfunction, *CHF* congestive heart failure, *SMR* standardised mortality ratio

**Fig. 2:**
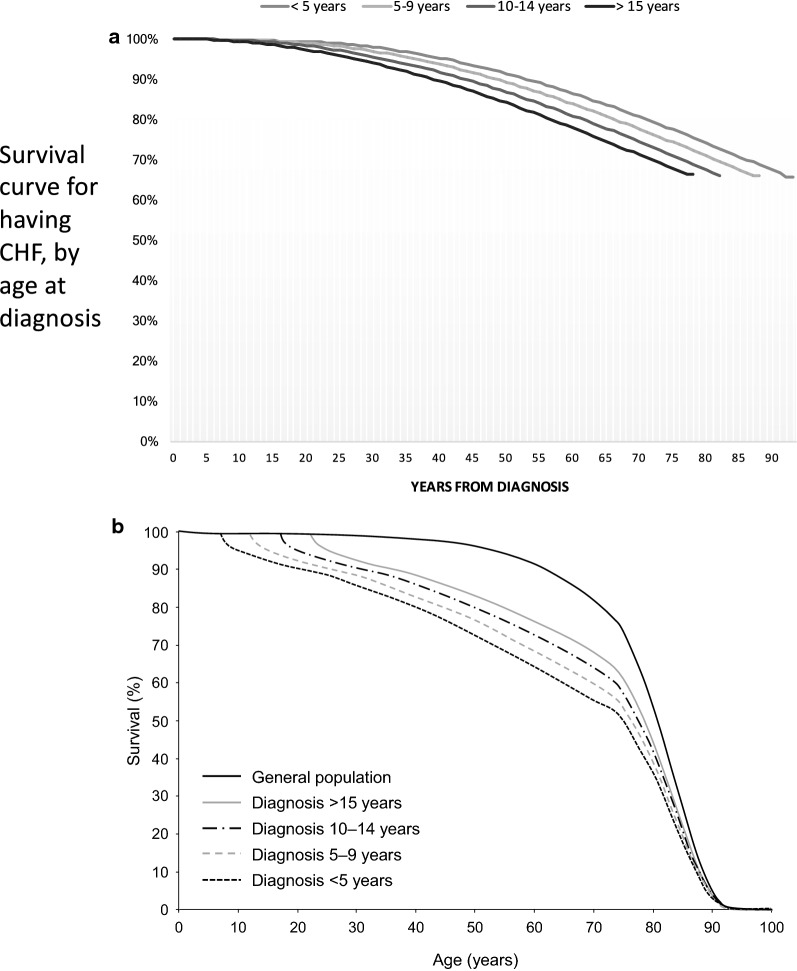
**a**, **b** Survival curves for paediatric patients who have not been treated with dexrazoxane who **a** have CHF, by age at diagnosis, and **b** are cancer survivors, conditional on surviving until the age at cancer diagnosis, compared to survival among the general population

Risk of death calculations included: death from recurrence or progression of the original cancer, developing a secondary malignancy, cardiac death, pulmonary death, death from external causes (accidents and violence), and death from other causes including infection, other diseases, and unknown cause. Risk of death by different causes was modelled using cause-specific cumulative mortality curves in the CCSS and sex-matched US population produced by Mertens et al. (see Fig. 2a and b) [20]. For the sensitivity analysis, the risk of death from different causes was modelled using national life tables for death, by age and gender [24], and distribution by cause of death for 5-year survivors of childhood cancer in France and the UK, as reported by Tukenova et al. [21]. A standardised mortality ratio (SMR) for childhood cancer survivors versus the general population was applied, based on findings that patients have a higher risk of cardiac mortality if they have ALVD or CHF [25].

In the current model, the treatment effect of dexrazoxane was based on relative risks from a published meta-analysis of randomised and non-randomised dexrazoxane studies of sarcoma and haematological malignancies, which evaluated the impact of adding dexrazoxane to anthracycline-based chemotherapy on the incidence of ‘clinical’ cardiotoxicity (CHF) and ‘clinical plus subclinical’ (ALVD) cardiotoxicity [26]. The Mantel–Haenszel (M–H) method was used to calculate the treatment effect of dexrazoxane in the base case, and the Bayesian approach was used in the sensitivity analyses (see Additional file 2).

### Cost inputs

The model programmed each patient’s treatment pathway and summed all costs and benefits accrued over the patient’s lifetime. Time spent in each health state (healthy, with ALVD, or with CHF), costs and utilities assigned to each health state, and total costs and outcomes were calculated.

The following cost parameters were included: drug and administration costs of dexrazoxane therapy; costs of heart failure diagnosis; heart failure treatment and management; heart failure hospitalisation; cardiac death; death from cancer; and death from other causes. National costs data were accessed from publicly available sources (see Additional files 3 and 4); where individual national healthcare costs systems did not match the model structure precisely, e.g. Diagnosis-Related Groups (DRG) costs in Germany, the model was either adapted to allow for the difference or approximations, based upon the available data, were made to allow for the design discrepancies (see Additional file 4). Where relevant, costs and benefits were discounted by 3.5% per annum, which is the standard reference rate in many European countries [27].

Utility data, differentiated by age and health state (healthy, with ALVD, or with CHF), were included in the model to enable the conversion of life years to quality-adjusted life years (QALYs) (see Additional file 5) [22]. In the sensitivity analyses, utility values were based on the New York Hear Association classes I and III, representing the health states ALVD and CHF, respectively [23].

### Model analyses

The primary outcome of the model was an estimation of total costs and benefits per patient, and the incremental costs and benefits incurred when receiving dexrazoxane. The incremental cost-effectiveness ratio (ICER) for using dexrazoxane was calculated.

Secondary outcomes of the model included the number of patients developing ALVD, the number of patients developing CHF, and the proportion of patients dying from either recurrence of the original cancer, cardiac illness, pulmonary reasons, or other causes.

Sensitivity analyses were undertaken using different data sources and predictive approaches for long-term outcomes (e.g. risk of developing CHF) as indicated in Table 1.

## Results

### Meta-analysis results

Five non-randomised studies, and one randomised study, of dexrazoxane in paediatric patients with sarcoma were identified from the literature [28–33]. These studies comprised 126 patients who received dexrazoxane and 154 patients in comparator groups. A further five paediatric studies (three randomised and two non-randomised studies) were identified involving a total of 606 patients with haematological malignancies who received dexrazoxane and 536 patients in the comparator groups [15, 33–37]. In these studies, acute lymphoblastic leukaemia and non-Hodgkin lymphoma were the most prevalent cancer types. Two further non-randomised studies reported outcomes data combining patients who had received anthracycline treatment for either sarcoma or haematological malignancy [38, 39]. Outcomes for the 176 patients who had received dexrazoxane and the 190 patients in the comparator group in these two studies were included in the meta-analysis for each tumour type.

The results of the meta-analyses were similar irrespective of the approach used (M–H or Bayesian). For patients with haematological malignancies, the relative risk (RR) of a cardiovascular event after dexrazoxane treatment compared to controls was 0.137 (95% confidence interval [CI] 0.032 to 0.582) when estimated using the M–H approach and 0.107 (95% CI 0.026 to 0.444) using the Bayesian approach. The equivalent RR for patients receiving anthracycline treatment for sarcoma were 0.188 (95% CI 0.072 to 0.494) and 0.219 (95% CI 0.086 to 0.558), respectively. Tests for the homogeneity of RR were not statistically significant for either tumour indication, demonstrating that the results are valid. The results presented are those calculated using the M–H approach for RR, model results for the Bayesian RR outcomes are included in Additional file 2.

### Model results

The additional duration of lifespan conferred by dexrazoxane treatment was small, amounting to approximately 0.02 life years per treated patient, irrespective of anthracycline dose administered or underlying indication for treatment. Dexrazoxane treatment resulted in a mean QALY benefit across the five countries ranging from 0.530 to 0.683 per dexrazoxane-treated patient. Dexrazoxane treatment conferred increasing benefits in terms of QALYs according to the total dose of anthracycline received with a mean of 0.530 QALY across all five countries for sarcoma patients receiving any anthracycline dose increasing to a mean of 0.596 QALY for sarcoma patients who received more than 250 mg/m^2^ anthracycline. The mean QALY gains for patients with haematological malignancies were 0.592 and 0.683, respectively. Model outcomes for lifespan and QALY were similar across all five countries, likely reflecting broadly comparable overall health outcomes and survival in each setting. Example results for the base case for France for all anthracycline doses are presented in Table 2. Results for all countries and anthracycline doses included in the analysis can be seen in Additional file 6.

**Table 2:** Base case results for France, all anthracycline doses

	Sarcoma patients		Haematological malignancy patients	
	Usual treatment	Usual treatment + dexrazoxane	Usual treatment	Usual treatment + dexrazoxane
Clinical events				
Proportion with CHF (%)	21.90	5.18	24.38	4.26
Average age at CHF diagnosis (years)	57.44	59.66	56.57	59.06
Years of life with CHF	4.90	1.07	5.45	0.88
Number of CHF hospitalizations	0.61	0.17	0.68	0.15
Average age at death (years)	68.93	69.00	67.42	67.55
Cause of death (%)				
Death from cancer	56.38	57.07	56.12	57.04
Cardiac death	5.42	4.12	5.69	3.95
Death from infection or respiratory disease	2.62	2.70	2.60	2.71
Death from other disease	28.06	28.49	28.10	28.69
Violent death	7.52	7.62	7.49	7.61
Death from unknown cause	0.00	0.00	0.00	0.00
QALYs				
QALYs without cardiac disease	18.379	19.593	18.528	20.099
QALYs with ALVD	1.044	0.681	1.094	0.500
QALYs with CHF	0.361	0.074	0.368	0.056
Total QALYs	19.783	20.349	19.990	20.654
Total LY disc	21.901	21.913	22.021	22.041
Costs (€)				
Drug and administration costs	7080.96	8123.61	7080.96	8123.61
Heart failure costs	757.48	199.24	806.93	140.74
Death costs	4261.18	4252.88	4198.35	4184.70
Total costs	12,099.62	12,575.73	12,086.25	12,449.05
Incrementals				
QALYs	0.565		0.665	
Costs (€)	476.11		362.80	
ICER (€)	894.55		545.87	

*ALVD* asymptomatic left ventricular dysfunction, *CHF* congestive heart failure, *ICER* incremental cost effectiveness ratio, *QALY* quality-adjusted life years

Overall, healthcare costs increased with increasing anthracycline dose, however, the additional costs due to dexrazoxane administration were offset at least in part by reductions in costs associated with cardiac failure and death. In two countries, Italy and Spain, these savings exceeded the acquisition cost of dexrazoxane by (€70–110) according to the dose of anthracycline administered. The highest additional costs of treatment due to dexrazoxane administration were in the UK at approximately €1464 (£1273^1^) for sarcoma patients receiving > 250 mg/m^2^ of doxorubicin or €1210 (£1052^1^) if any total dose of doxorubicin was considered; costs for patients with haematological malignancies were slightly lower at €1281 and €1083, respectively.

The ICER for dexrazoxane treatment was favourable in all countries irrespective of anthracycline dose for both sarcoma and haematological malignancies. Given the differences in healthcare costs across different healthcare systems, the ICER varied marginally between countries (range: dominant to €2196) with the highest ICER reported in Germany and the UK and lowest in Spain and Italy (see Table 3 and Additional file 6). Interestingly, the ICER increased with anthracycline dose for both patients with sarcoma and patients with haematological malignancies.

**Table 3:** ICER for dexrazoxane administration according to anthracycline dose treatment of sarcoma and haematological malignancies

Malignancy type	France (€)	Germany (€)	UK (£)^a^	Italy (€)	Spain (€)
Sarcoma					
All AC doses	895	2006	1983	Dominant	Dominant
> 100 mg/m^2^	891	1991	1986	Dominant	Dominant
> 250 mg/m^2^	982	2196	2135	Dominant	Dominant
Haematological					
All AC doses	559	1418	1590	Dominant	Dominant
> 100 mg/m^2^	563	1400	1579	Dominant	Dominant
> 250 mg/m^2^	586	1462	1630	Dominant	Dominant

^a^£ sterling amounts converted to Euro at a rate of 1.15€ to one £ for calculations of mean values

*AC* anthracycline, *ICER* incremental cost effectiveness ratio

### Sensitivity analyses

Sensitivity analyses using alternative data sources and predictive approaches for long-term outcomes as indicated in Table 1 produced similar results to those reported for the base case above. The results for the one-way sensitivity analyses for France (example case) are presented in Table 4.

**Table 4:** One-way sensitivity analyses for sarcoma patients in France, all anthracycline doses

Description of analysis	No dexrazoxane		With dexrazoxane		Incremental costs €	Incremental QALYs	ICER €
	Total costs €	Total QALYs	Total costs €	Total QALYs			
Base case	12,102	19.78	12,549	20.35	447	0.57	791
Risk of CHF is based on French general population prevalence data multiplied with relative risks for childhood cancer survivors [15]	18,047	15.22	15,437	17.61	− 2609	2.39	Dominant
Risk of CHF is extrapolated with a linear function	11,854	20.06	12,477	20.48	624	0.42	1493
Risk of death modelled with general population life tables multiplied by SMR for childhood cancer survivors [20]	13,103	19.70	13,668	20.17	565	0.47	1214
Treatment effect modelled with Bayesian RR	12,099	19.78	12,575	20.31	476	0.53	895
Utility data based on NYHA class [22] and not differentiated by age	12,099	20.37	12,548	20.61	449	0.23	1922

*CHF* congestive heart failure, *ICER* incremental cost effectiveness ratio, *NYHA* New York Heart Association, *QALY* quality-adjusted life years, *RR* relative risk, *SMR* standardised mortality ratio

## Discussion

The results presented here indicate that dexrazoxane is a highly cost-effective therapy for the prevention of anthracycline cardiotoxicity in paediatric cancer patients. Few pharmacologic interventions offer such favourable ICER in the field of oncology and these results highlight the need for cardiologists and paediatric oncologists to take a multidisciplinary approach to optimise patient care in the face of cost-effective treatment options.

The highest anthracycline dose category in this model was arbitrarily set at > 250 mg/m^2^ since this reflected dose sub-group analyses in the clinical studies upon which the model is based. The results of the model can be anticipated to reflect expected outcomes in patients receiving > 300 mg/m^2^ anthracycline since the differences in model outcomes between anthracycline dose category are relatively small. Indeed, the model supports the use of dexrazoxane as recommended when the expected dose to be administered exceeds the 300 mg/m^2^ anthracycline threshold as it demonstrates cost effectiveness irrespective of the actual anthracycline dose administered.

Current management of haematological malignancies in childhood typically involves administration of anthracycline doses as high as 250 mg/m^2^. However, most recommended regimens limit anthracycline exposure to around 100 mg/m^2^. Doses exceeding 250 mg/m^2^ are often used for acute myeloid leukaemia and lymphomas [40]. Our core model only considered doses above 100 mg/m^2^ since the lifetime risk of cardiotoxicity is much lower below it. Extrapolation of the results at higher dose levels and the results for all doses of anthracyclines suggest dexrazoxane treatment is likely to be cost-effective at lower anthracycline doses but this needs to be considered in the light of limited efficacy and safety data for the > 250 mg/m^2^ dose level.

This model relies upon the assumption that differences in the proportions of patients observed to have left ventricular dysfunction based upon arbitrary echocardiographic outcomes during anthracycline treatment will translate into differences in long term cardiac outcomes. This hypothesis cannot be tested but it is supported by long term observational data amongst patients treated with dexrazoxane or placebo in COG trials [41]. At a mean of 16 years after initial diagnosis, dexrazoxane-treated patients had significantly higher left ventricular ejection fraction overall compared with controls (those who did not receive dexrazoxane), with significantly higher myocardial wall stress and dysfunction reported in the subset of patients from one study (COG P9404) who had received 360 mg/m^2^ of doxorubicin. An additional weakness of this model is the inclusion of studies where dexrazoxane was administered at a dose of 20:1 the doxorubicin dose in the meta-analyses of treatment effect. Whilst a 20:1 dose regimen was considered to have poorer safety profile than the 10:1 regimen, leading to its withdrawal, there are little data available to suggest it was significantly more efficacious. Therefore, for the purposes of constructing this model the advantages of including the additional volume of efficacy data were considered to outweigh the disadvantages of potential variations in cardioprotective benefit. Importantly, these modelled analyses assume no impact of dexrazoxane upon the incidence of SMN, nor do they include any allowance for improved oncologic outcomes that might be a consequence of dexrazoxane administration.

Although this model attempts to characterise the costs associated for each of the five countries reported, comparisons across countries should be treated with caution. Individual country data for the costs of investigations, and scope of costs related to in-patient and out-patient management were accessed from local sources. However, because there are differences in the way healthcare costs are calculated within each system, especially for those associated with the inpatient management of heart failure, comparisons are not on a fully like for like basis. This may explain in part the higher ICER reported for Germany and the UK but differences in medications acquisition costs are also relevant.

## Conclusions

In conclusion, dexrazoxane is a highly cost-effective treatment for prevention of anthracycline related cardiotoxicity in paediatric patients with sarcoma and haematological malignancies. In two countries, Spain and Italy, the model demonstrates dominance for dexrazoxane in terms of ICER, whilst in the remaining three major European countries all model outcomes demonstrate an ICER less than one tenth of the recognised standard threshold for cost-effectiveness of £20,000–£30,000 set by NICE [42]. There should therefore be no economic barrier to the use of dexrazoxane for the prevention of anthracycline related cardiotoxicity in paediatric patients with sarcoma and haematological malignancies in Europe.

## Supplementary information

**Additional file 1.** Meta-analysis search strategy. Table showing the search terms and filters utilised in the literature search.

**Additional file 2.** Relative risks for clinical cardiotoxicity and subclinical toxicity. Table showing the relative risk data for clinical cardiotoxicity and subclinical cardiotoxicity based on standardised mortality ratio evidence only or based on non-randomised, double-arm interventional studies.

**Additional file 3.** Sources for national specific healthcare costs used. Table showing the sources used for each input variable, with references, for the specific healthcare costs for each of the five included countries.

**Additional file 4.** Summary of healthcare cost data sources. Table showing a summary of healthcare cost data sources.

**Additional file 5.** Utilities by age and health state. Table showing details of the utility data, by age and health state, included in the model.

**Additional file 6.** Base case results per country, all anthracycline doses. Table showing the base case results for Germany, Italy, Spain and the United Kingdom.

## Data Availability

The datasets used and/or analysed during the current study are available from the corresponding author on reasonable request.

## Abbreviations

ALVD: Asymptomatic left ventricular dysfunction
CCSS: Childhood Cancer Survivor Study
CHF: Congestive heart failure
CI: Confidence interval
COG: Children’s Oncology Group
DRG: Diagnosis-Related Groups
ICER: Incremental cost-effectiveness ratio
M–H: Mantel–Haenszel
QALY: Quality-adjusted life years
QoL: Quality of life
RCT: Randomised clinical trial
RR: Relative risk
SMN: Second malignant neoplasm
SMR: Standardised mortality ratio
UK: United Kingdom
US: United States

## Footnotes

1 Euro amount calculated at €1.15 to 1GBP.
