# Supplementary material for: Evaluation of the cost-effectiveness of dexrazoxane for the prevention of anthracycline-related cardiotoxicity in children with sarcoma and haematologic malignancies: a European perspective
Source: Cost Eff Resour Alloc. 2020 Feb 10;18:7. doi: 10.1186/s12962-020-0205-4 (PMC7011276; doi:10.1186/s12962-020-0205-4)
Supplement: Supplementary file 1 — Additional file 1. Meta-analysis search strategy. Table showing the search terms and filters utilised in the literature search. [file 12962_2020_205_MOESM1_ESM.docx]

**Additional File 1. Meta-analysis search strategy.**

Identification of published clinical trials evaluating the addition of dexrazoxane to anthracycline therapy in the treatment of childhood cancer

| **Search Terms** | **Hits** | **Excluded** |
| --- | --- | --- |
| dexrazoxane AND anthracycline AND cancer AND (paediatric OR children) AND Clinical Trial[ptyp] | 19 | 8 |
