# Supplementary material for: Evaluation of the cost-effectiveness of dexrazoxane for the prevention of anthracycline-related cardiotoxicity in children with sarcoma and haematologic malignancies: a European perspective
Source: Cost Eff Resour Alloc. 2020 Feb 10;18:7. doi: 10.1186/s12962-020-0205-4 (PMC7011276; doi:10.1186/s12962-020-0205-4)
Supplement: Supplementary file 2 — Additional file 2. Relative risks for clinical cardiotoxicity and subclinical toxicity. Table showing the relative risk data for clinical cardiotoxicity and subclinical cardiotoxicity based on standardised mortality ratio evidence only or based on non-randomised, double-arm interventional studies. [file 12962_2020_205_MOESM2_ESM.docx]

**Additional File 2. Relative risks for clinical cardiotoxicity and subclinical toxicity.**

| **Outcome measure** | **Based on SMR evidence only** | **Based on non-randomised, double-arm interventional studies** |
| --- | --- | --- |
| Clinical cardiotoxicity | - RR=0.24 (0.03; 2.09) p=0.20   (only three events observed in four studies with 991 pts) | - RR=0.29 (0.14; 0.61) p=0.001*   (42 events observed in eight studies with 741 patients) |
| Clinical + subclinical cardiotoxicity | - RR=0.29 (0.13; 0.64) p=0.003*   (22 events observed in four studies with 990 patients) | - RR=0.43 (0.30; 0.63) p<0.00001*   (99 events observed in eight studies with 521 patients) |

* Statistically significant

RCT, randomised clinical trial; RR, relative risk; SMR, standardised mortality ratio
