# Supplementary material for: Evaluation of the cost-effectiveness of dexrazoxane for the prevention of anthracycline-related cardiotoxicity in children with sarcoma and haematologic malignancies: a European perspective
Source: Cost Eff Resour Alloc. 2020 Feb 10;18:7. doi: 10.1186/s12962-020-0205-4 (PMC7011276; doi:10.1186/s12962-020-0205-4)
Supplement: Supplementary file 3 — Additional file 3. Sources for national specific healthcare costs used. Table showing the sources used for each input variable, with references, for the specific healthcare costs for each of the five included countries. [file 12962_2020_205_MOESM3_ESM.docx]

**Additional File 3. Sources for national specific healthcare costs used.**

| **Input variable** | **France** | **Germany*** | **UK** | **Spain** | **Italy*** |
| --- | --- | --- | --- | --- | --- |
| Non-medication resources |  | Mean value for other markets | NHS UK Reference costs [1] | eSalud [2] | Emilia Romagna Region [4] |
| Physician fees |  | <https://bookimed.com/clinics/country=germany/direction=cardiology> | NHS UK Reference costs [1] |  |  |
| Cardioxane costs | Clinigen Group PLC | Clinigen Group PLC | Clinigen Group PLC | Clinigen Group PLC | Clinigen Group PLC |
| Other medication costs |  | German drug prices database | BNF Online accessed 13/8/18 | <https://botplusweb.portalfarma.com3> | Farmadati [5] |
| In-patient hospital costs for CHF |  | G-DRG Catalogue | NHS UK Reference costs [1] | Ministerio de Sanidad, Consumo y Bienestar Social. Instituto de Información Sanitaria.[3] | Emilia Romagna Region [4] |
| Investigations |  | <https://www.dguv.de/medien/inhalt/reha_leistung/verguetung/uv-goae.pdf> | National average cost for directly accessed pathology services 2017-18 [1] | eSalud [2] | Italy: Emilia Romagna Region [6] |

* Note for some variables, costs were not available in individual markets and a mean value was taken across the information available in other markets

### References

1. NHS: **NHS Reference Costs 2017 to 2018** [https://improvement.nhs.uk/documents/1972/1_-_Reference_costs_201718.pdf] Accessed 28 November 2018.
2. Gisbert R, Brosa M: **eSalud - Información económica del sector sanitario** [http://www.oblikue.com/bddcostes/] Accessed 19 November 2018.
3. Ministerio de Sanidad Consumo y Bienestar Social: **Consulta Interactiva del Sistema Nacional de Salud** [https://pestadistico.inteligenciadegestion.mscbs.es/PUBLICOSNS/Comun/DefaultPublico.aspx] Accessed 20 November 2018.
4. Ministero della Salute: **Tavole Rapporto SDO 2016** [http://www.salute.gov.it/portale/documentazione/p6_2_8_3_1.jsp?lingua=italiano&id=28] Accessed 04 September 2018.
5. FarmadatiItalia: **Banche Dati del Farmaco Parafarmaco e Dispositivo Medico** [https://www.farmadati.it/] Accessed 04 September 2018.
6. Federlab Italia: **Tariffe di laboratorio: Analisi comparativa dei Tariffari Regionali e Ministeriali.** [http://www.federlabitalia.com/documenti_110609_2.htm] Accessed 04 September 2018.
