# Supplementary material for: Evaluation of the cost-effectiveness of dexrazoxane for the prevention of anthracycline-related cardiotoxicity in children with sarcoma and haematologic malignancies: a European perspective
Source: Cost Eff Resour Alloc. 2020 Feb 10;18:7. doi: 10.1186/s12962-020-0205-4 (PMC7011276; doi:10.1186/s12962-020-0205-4)
Supplement: Supplementary file 4 — Additional file 4. Summary of healthcare cost data sources. Table showing a summary of healthcare cost data sources. [file 12962_2020_205_MOESM4_ESM.docx]

**Additional File 4. Summary of healthcare cost data sources.**

Healthcare costs were divided into five categories, with a large number of variables included in each category to provide the basis for cost of management of patients developing cardiac failure. Individual tariffs were sourced wherever possible for specific procedures as summarised below:

- Procedures and Investigations (n=17)
  - e.g. 12 lead ECG, Functional MRI, Cardiologist consultant
- Oncology Medicines Costs
  - Doxorubicin, dexrazoaxne
- Cardiology Medicines Costs (n=22)
  - ACE inhibitors and beta blockers at various doses
- Blood Tests (n=26)
  - Clinical chemistry and haematology, individual or panel tests
- Hospital stay costs
  - Weighted mean tariff based on reference costs for differing heart failure severity and numbers of episodes reported

Individual country data were identified from local sources as summarised below. There was considerable variation in the level of detail available for different aspects of healthcare costs across different countries. Where no data were available, an appropriate substitution was made. For example, materials costs for blood tests are not available within the German DRG system and these were replaced by the amount that a physician may charge was utilised. In contrast, for the UK the national average costs for directly accessed pathology services were used to inform the model. For Spain, healthcare is decentralised and an average value was calculated based upon tariffs for each region or Autonomous Community.

**Costs data sources.**

|  | **Source** | **Date Accessed** |
| --- | --- | --- |
| **Procedures and Investigations** |  |  |
| United Kingdom | https://www.gov.uk/government/publications/nhs-reference-costs-2015-to-2016 | 23/08/18 |
| France | http://base-donnees-publique.medicaments.gouv.fr | 05/11/18 |
| Germany | https://bookimed.com/clinics/country=germany/direction=cardiology/ | 28/08/18 |
| Spain | Gisbert, R and Brosa, M. Spanish Health Costs and cost-effectiveness ratios Database: eSalud [Internet]. Barcelona: Oblikue Consulting, S.L. http://www.oblikue.com/bddcostes/. | 19/11/18 |
| Italy | http://www.salute.gov.it/portale/documentazione/p6_2_8_3_1.jsp?lingua=italiano&id=28 | 04/09/18 |
| **Oncology medicines** |  |  |
| United Kingdom | www.bnf.org | 13/08/18 |
| France | http://base-donnees-publique.medicaments.gouv.fr | 05/11/18 |
| Germany | Clinigen PLC | NA |
| Spain | Con. Gen. de Col. Oficiales de Farmacéuticos. https://botplusweb.portalfarma.com | 20/11/18 |
| Italy | www.farmadati.it | 28/09/18 |
| **Cardiology medicines** |  |  |
| United Kingdom | www.bnf.org | 13/08/18 |
| France | http://base-donnees-publique.medicaments.gouv.fr | 05/11/18 |
| Germany | www.pharmazie.com | 21/08/18 |
| Spain | Con. Gen. de Col. Oficiales de Farmacéuticos. https://botplusweb.portalfarma.com | 20/11/18 |
| Italy | www.farmadati.it | 28/09/18 |
| **Blood tests and Hospital stay costs** |  |  |
| United Kingdom | https://www.gov.uk/government/publications/nhs-reference-costs-2015-to-2016 | 23/08/18 |
| France | http://base-donnees-publique.medicaments.gouv.fr | 05/11/18 |
| Germany | https://www.dguv.de/medien/inhalt/reha_leistung/verguetung/uv-goae.pdf | 21/08/18 |
| Spain | Ministerio de Sanidad, Consumo y Bienestar Social. Instituto de Información Sanitaria. Registro de altas – CMBD. http://pestadistico.inteligenciadegestion.msssi.es/ | 22/11/18 |
| Italy | http://www.federlabitalia.com/documenti_110609_2.htm | 04/09/18 |
