# Supplementary material for: Evaluation of the cost-effectiveness of dexrazoxane for the prevention of anthracycline-related cardiotoxicity in children with sarcoma and haematologic malignancies: a European perspective
Source: Cost Eff Resour Alloc. 2020 Feb 10;18:7. doi: 10.1186/s12962-020-0205-4 (PMC7011276; doi:10.1186/s12962-020-0205-4)
Supplement: Supplementary file 5 — Additional file 5. Utilities by age and health state. Table showing details of the utility data, by age and health state, included in the model. [file 12962_2020_205_MOESM5_ESM.docx]

**Additional File 5. Utilities by age and health state [1].**

| **Health state** | **Age <26** | **Age 26–45** | **Age 46–65** | **Age >65** |
| --- | --- | --- | --- | --- |
| No ALVD | 0.980 | 0.947 | 0.913 | 0.860 |
| With ALVD | 0.960 | 0.834 | 0.697 | 0.510 |
| With heart failure | 0.500 | 0.427 | 0.366 | 0.270 |

ALVD, asymptomatic left ventricular dysfunction

### Reference

1. Wong FL, Bhatia S, Landier W, Francisco L, Leisenring W, Hudson MM, Armstrong GT, Mertens A, Stovall M, Robison LL, et al: **Cost-effectiveness of the children's oncology group long-term follow-up screening guidelines for childhood cancer survivors at risk for treatment-related heart failure.** *Ann Intern Med* 2014, **160:**672-683.
