# Supplementary material for: Evaluation of the cost-effectiveness of dexrazoxane for the prevention of anthracycline-related cardiotoxicity in children with sarcoma and haematologic malignancies: a European perspective
Source: Cost Eff Resour Alloc. 2020 Feb 10;18:7. doi: 10.1186/s12962-020-0205-4 (PMC7011276; doi:10.1186/s12962-020-0205-4)
Supplement: Supplementary file 6 — Additional file 6. Base case results per country, all anthracycline doses. Table showing the base case results for Germany, Italy, Spain and the United Kingdom. [file 12962_2020_205_MOESM6_ESM.docx]

**Additional File 6. Base Case results per country, all anthracycline doses**

**A: Base Case results for France**

|  | **All anthracycline doses** | | | | **Anthracycline >100 mg/m^2^** | | | | **Anthracycline >250 mg/m^2^** | | | |
| --- | --- | --- | --- | --- | --- | --- | --- | --- | --- | --- | --- | --- |
|  | **Sarcoma patients** | | **Haematological malignancy patients** | | **Sarcoma** | | **Haematological malignancy patients** | | **Sarcoma** | | **Haematological malignancy patients** | |
|  | **Usual treatment** | **Usual treatment + dexrazoxane** | **Usual treatment** | **Usual treatment + dexrazoxane** | **Usual treatment** | **Usual treatment + dexrazoxane** | **Usual treatment** | **Usual treatment + dexrazoxane** | **Usual treatment** | **Usual treatment + dexrazoxane** | **Usual treatment** | **Usual treatment + dexrazoxane** |
| **Clinical events** |  |  |  |  |  |  |  |  |  |  |  |  |
| Proportion with CHF (%) | 21.90 | 5.18 | 24.38 | 4.26 | 23.54 | 5.58 | 26.10 | 3.57 | 24.83 | 6.97 | 28.17 | 3.81 |
| Average age at CHF diagnosis (years) | 57.44 | 59.66 | 56.57 | 59.06 | 57.40 | 59.71 | 56.48 | 59.03 | 57.12 | 59.91 | 55.99 | 58.90 |
| Years of life with CHF | 4.90 | 1.07 | 5.45 | 0.88 | 5.25 | 1.15 | 5.86 | 0.74 | 5.59 | 1.41 | 6.42 | 0.79 |
| Number of CHF hospitalizations | 0.61 | 0.17 | 0.68 | 0.15 | 0.65 | 0.18 | 0.73 | 0.13 | 0.69 | 0.22 | 0.78 | 0.14 |
| Average age at death (years) | 68.93 | 69.00 | 67.42 | 67.55 | 68.92 | 69.00 | 67.41 | 67.54 | 68.91 | 68.99 | 67.38 | 67.54 |
| **Cause of death (%)** |  |  |  |  |  |  |  |  |  |  |  |  |
| Death from cancer | 56.38 | 57.07 | 56.12 | 57.04 | 56.30 | 57.05 | 56.07 | 57.04 | 56.26 | 57.01 | 55.97 | 57.04 |
| Cardiac death | 5.42 | 4.12 | 5.69 | 3.95 | 5.57 | 4.14 | 5.80 | 3.94 | 5.69 | 4.22 | 6.00 | 3.95 |
| Death from infection or respiratory disease | 2.62 | 2.70 | 2.60 | 2.71 | 2.62 | 2.70 | 2.59 | 2.74 | 2.59 | 2.69 | 2.63 | 2.74 |
| Death from other disease | 28.06 | 28.49 | 28.10 | 28.69 | 27.99 | 28.50 | 28.05 | 28.67 | 27.94 | 28.48 | 27.92 | 28.66 |
| Violent death | 7.52 | 7.62 | 7.49 | 7.61 | 7.52 | 7.61 | 7.49 | 7.61 | 7.52 | 7.60 | 7.48 | 7.61 |
| Death from unknown cause | 0.00 | 0.00 | 0.00 | 0.00 | 0.00 | 0.00 | 0.00 | 0.00 | 0.00 | 0.00 | 0.00 | 0.00 |
| **QALYs** |  |  |  |  |  |  |  |  |  |  |  |  |
| QALYs without cardiac disease | 18.379 | 19.593 | 18.528 | 20.099 | 18.211 | 19.419 | 18.355 | 19.678 | 18.078 | 19.342 | 18.149 | 19.565 |
| QALYs with ALVD | 1.044 | 0.681 | 1.094 | 0.500 | 1.116 | 0.733 | 1.171 | 0.835 | 1.175 | 0.823 | 1.260 | 0.915 |
| QALYs with CHF | 0.361 | 0.074 | 0.368 | 0.056 | 0.388 | 0.079 | 0.396 | 0.046 | 0.411 | 0.097 | 0.434 | 0.049 |
| Total QALYs | 19.783 | 20.349 | 19.990 | 20.654 | 19.716 | 20.322 | 19.922 | 20.559 | 19.663 | 20.262 | 19.844 | 20.529 |
| Total LY disc | 21.901 | 21.913 | 22.021 | 22.041 | 21.900 | 21.913 | 22.019 | 22.041 | 21.898 | 21.912 | 22.015 | 22.040 |
| **Costs (€)** |  |  |  |  |  |  |  |  |  |  |  |  |
| Drug and administration costs | 7080.96 | 8123.61 | 7080.96 | 8123.61 | 7522.90 | 8640.41 | 7522.89 | 8640.41 | 8240.10 | 9477.70 | 8240.10 | 9477.70 |
| Heart failure costs | 757.48 | 199.24 | 806.93 | 140.74 | 813.13 | 213.27 | 861.18 | 117.72 | 865.75 | 225.56 | 945.71 | 126.93 |
| Death costs | 4261.18 | 4252.88 | 4198.35 | 4184.70 | 4265.08 | 4256.08 | 4201.24 | 4186.29 | 4267.88 | 4258.22 | 4208.51 | 4191.48 |
| Total costs | 12099.62 | 12575.73 | 12086.25 | 12449.05 | 12606.39 | 13109.76 | 12595.32 | 12944.43 | 13373.73 | 13961.48 | 13398.83 | 13832.61 |
| **Incrementals** |  |  |  |  |  |  |  |  |  |  |  |  |
| QALYs | 0.565 |  | 0.665 |  | 0.571 |  | 0.637 |  | 0.599 |  | 0.685 |  |
| Costs (€) | 476.11 |  | 362.80 |  | 508.66 |  | 359.11 |  | 587.75 |  | 401.79 |  |
| ICER (€) | 894.55 |  | 545.87 |  | 890.96 |  | 563.58 |  | 981.83 |  | 585.83 |  |

ALVD, asymptomatic left ventricular dysfunction; CHF, congestive heart failure; ICER, incremental cost effectiveness ratio; QALY, quality-adjusted life years

**B: Base Case results for Germany**

|  | **All anthracycline doses** | | | | **Anthracycline >100 mg/m^2^** | | | | **Anthracycline >250 mg/m^2^** | | | |
| --- | --- | --- | --- | --- | --- | --- | --- | --- | --- | --- | --- | --- |
|  | **Sarcoma patients** | | **Haematological malignancy patients** | | **Sarcoma** | | **Haematological malignancy patients** | | **Sarcoma** | | **Haematological malignancy patients** | |
|  | **Usual treatment** | **Usual treatment + dexrazoxane** | **Usual treatment** | **Usual treatment + dexrazoxane** | **Usual treatment** | **Usual treatment + dexrazoxane** | **Usual treatment** | **Usual treatment + dexrazoxane** | **Usual treatment** | **Usual treatment + dexrazoxane** | **Usual treatment** | **Usual treatment + dexrazoxane** |
| **Clinical events** |  |  |  |  |  |  |  |  |  |  |  |  |
| Proportion with CHF (%) | 21.45 | 5.91 | 23.95 | 3.20 | 23.08 | 6.38 | 25.63 | 3.50 | 24.38 | 6.78 | 27.68 | 3.74 |
| Average age at CHF diagnosis (years) | 56.97 | 59.40 | 56.18 | 58.28 | 56.97 | 59.43 | 56.07 | 58.65 | 56.72 | 59.39 | 55.60 | 58.53 |
| Years of life with CHF | 4.75 | 1.20 | 5.30 | 0.68 | 5.09 | 1.29 | 5.69 | 0.72 | 5.42 | 1.37 | 6.24 | 0.77 |
| Number of CHF hospitalizations | 0.59 | 0.19 | 0.65 | 0.12 | 0.64 | 0.20 | 0.70 | 0.13 | 0.68 | 0.21 | 0.78 | 0.14 |
| Average age at death (years) | 68.34 | 68.40 | 66.88 | 66.99 | 68.34 | 68.41 | 66.87 | 66.99 | 68.33 | 68.40 | 66.85 | 66.99 |
| **Cause of death (%)** |  |  |  |  |  |  |  |  |  |  |  |  |
| Death from cancer | 56.42 | 57.08 | 56.14 | 57.08 | 56.32 | 57.05 | 56.08 | 57.07 | 56.29 | 57.04 | 55.99 | 57.06 |
| Cardiac death | 5.48 | 4.27 | 5.75 | 3.97 | 5.64 | 4.31 | 5.86 | 4.00 | 5.75 | 4.33 | 6.05 | 4.02 |
| Death from infection or respiratory disease | 2.60 | 2.63 | 2.56 | 2.71 | 2.60 | 2.63 | 2.55 | 2.69 | 2.57 | 2.62 | 2.59 | 2.69 |
| Death from other disease | 27.98 | 28.40 | 28.06 | 28.62 | 27.92 | 28.40 | 28.02 | 28.63 | 27.87 | 28.40 | 27.89 | 28.62 |
| Violent death | 7.52 | 7.62 | 7.49 | 7.62 | 7.52 | 7.61 | 7.49 | 7.61 | 7.52 | 7.61 | 7.48 | 7.61 |
| Death from unknown cause | 0.00 | 0.00 | 0.00 | 0.00 | 0.00 | 0.00 | 0.00 | 0.00 | 0.00 | 0.00 | 0.00 | 0.00 |
| **QALYs** |  |  |  |  |  |  |  |  |  |  |  |  |
| QALYs without cardiac disease | 18.349 | 19.465 | 18.503 | 19.729 | 18.185 | 19.377 | 18.332 | 19.642 | 18.052 | 19.301 | 18.128 | 19.530 |
| QALYs with ALVD | 1.033 | 0.710 | 1.084 | 0.764 | 1.104 | 0.765 | 1.161 | 0.825 | 1.163 | 0.813 | 1.250 | 0.904 |
| QALYs with CHF | 0.356 | 0.085 | 0.363 | 0.043 | 0.383 | 0.091 | 0.391 | 0.045 | 0.406 | 0.096 | 0.428 | 0.048 |
| Total QALYs | 19.738 | 20.261 | 19.952 | 20.536 | 19.672 | 20.233 | 19.885 | 20.512 | 19.621 | 20.209 | 19.808 | 20.483 |
| Total LY disc | 21.828 | 21.839 | 21.958 | 21.977 | 21.827 | 21.838 | 21.956 | 21.977 | 21.826 | 21.838 | 21.952 | 21.976 |
| **Costs (€)** |  |  |  |  |  |  |  |  |  |  |  |  |
| Drug and administration costs | 13319.88 | 15495.19 | 13319.88 | 15495.19 | 14177.72 | 16509.25 | 14177.72 | 16509.25 | 15569.89 | 18151.95 | 15569.89 | 18151.95 |
| Heart failure costs | 1490.55 | 371.65 | 1526.12 | 192.64 | 1604.24 | 397.68 | 1642.36 | 204.79 | 1699.17 | 418.12 | 1799.97 | 221.61 |
| Death costs | 4303.34 | 4295.86 | 4233.76 | 4221.02 | 4307.08 | 4298.87 | 4236.55 | 4222.51 | 4309.69 | 4300.93 | 4243.62 | 4227.52 |
| Total costs | 19113.77 | 20162.71 | 19079.76 | 19908.85 | 20087.04 | 21205.80 | 20058.63 | 20936.55 | 21578.75 | 22871.00 | 21613.48 | 22601.08 |
| **Incrementals** |  |  |  |  |  |  |  |  |  |  |  |  |
| QALYs | 0.523 |  | 0.585 |  | 0.561 |  | 0.627 |  | 0.588 |  | 0.675 |  |
| Costs (€) | 1048.93 |  | 829.09 |  | 1116.75 |  | 877.92 |  | 1292.26 |  | 987.60 |  |
| ICER (€) | 2006.01 |  | 1418.19 |  | 1991.30 |  | 1399.81 |  | 2196.76 |  | 1462.13 |  |

ALVD, asymptomatic left ventricular dysfunction; CHF, congestive heart failure; ICER, incremental cost effectiveness ratio; QALY, quality-adjusted life years

**B: Base Case results for Italy**

|  | **All anthracycline doses** | | | | **Anthracycline >100 mg/m^2^** | | | | **Anthracycline >250 mg/m^2^** | | | |
| --- | --- | --- | --- | --- | --- | --- | --- | --- | --- | --- | --- | --- |
|  | **Sarcoma patients** | | **Haematological malignancy patients** | | **Sarcoma** | | **Haematological malignancy patients** | | **Sarcoma** | | **Haematological malignancy patients** | |
|  | **Usual treatment** | **Usual treatment + dexrazoxane** | **Usual treatment** | **Usual treatment + dexrazoxane** | **Usual treatment** | **Usual treatment + dexrazoxane** | **Usual treatment** | **Usual treatment + dexrazoxane** | **Usual treatment** | **Usual treatment + dexrazoxane** | **Usual treatment** | **Usual treatment + dexrazoxane** |

| **Clinical events** |  |  |  |  |  |  |  |  |  |  |  |  |
| --- | --- | --- | --- | --- | --- | --- | --- | --- | --- | --- | --- | --- |
| Proportion with CHF (%) | 21.90 | 6.08 | 24.38 | 3.27 | 23.54 | 6.57 | 26.10 | 3.57 | 24.83 | 6.97 | 28.17 | 3.81 |
| Average age at CHF diagnosis (years) | 57.44 | 59.91 | 56.57 | 58.67 | 57.41 | 59.97 | 56.48 | 59.03 | 57.12 | 59.91 | 55.99 | 58.90 |
| Years of life with CHF | 4.90 | 1.24 | 5.45 | 0.69 | 5.25 | 1.33 | 5.83 | 0.74 | 5.59 | 1.41 | 6.42 | 0.79 |
| Number of CHF hospitalizations | 0.61 | 0.19 | 0.67 | 0.12 | 0.64 | 0.21 | 0.74 | 0.13 | 0.69 | 0.22 | 0.80 | 0.14 |
| Average age at death (years) | 68.93 | 69.00 | 67.42 | 67.55 | 68.93 | 69.00 | 67.41 | 67.55 | 68.92 | 69.00 | 67.39 | 67.54 |
| **Cause of death (%)** |  |  |  |  |  |  |  |  |  |  |  |  |
| Death from cancer | 56.38 | 57.05 | 56.12 | 57.06 | 56.30 | 57.02 | 56.07 | 57.04 | 56.26 | 57.01 | 55.97 | 57.04 |
| Cardiac death | 5.42 | 4.17 | 5.69 | 3.92 | 5.57 | 4.20 | 5.80 | 3.94 | 5.69 | 4.22 | 6.00 | 3.95 |
| Death from infection or respiratory disease | 2.61 | 2.68 | 2.59 | 2.73 | 2.61 | 2.69 | 2.58 | 2.73 | 2.58 | 2.68 | 2.62 | 2.73 |
| Death from other disease | 28.07 | 28.49 | 28.11 | 28.67 | 28.00 | 28.49 | 28.06 | 28.68 | 27.95 | 28.49 | 27.93 | 28.67 |
| Violent death | 7.52 | 7.61 | 7.49 | 7.62 | 7.52 | 7.60 | 7.49 | 7.61 | 7.52 | 7.60 | 7.48 | 7.61 |
| Death from unknown cause | 0.00 | 0.00 | 0.00 | 0.00 | 0.00 | 0.00 | 0.00 | 0.00 | 0.00 | 0.00 | 0.00 | 0.00 |
| **QALYs** |  |  |  |  |  |  |  |  |  |  |  |  |
| QALYs without cardiac disease | 18.380 | 19.510 | 18.529 | 19.768 | 18.212 | 19.420 | 18.355 | 19.679 | 18.078 | 19.343 | 18.150 | 19.566 |
| QALYs with ALVD | 1.044 | 0.720 | 1.093 | 0.773 | 1.116 | 0.775 | 1.171 | 0.835 | 1.175 | 0.823 | 1.261 | 0.915 |
| QALYs with CHF | 0.361 | 0.087 | 0.367 | 0.043 | 0.388 | 0.092 | 0.396 | 0.045 | 0.411 | 0.097 | 0.433 | 0.049 |
| Total QALYs | 19.784 | 20.317 | 19.990 | 20.584 | 19.716 | 20.288 | 19.923 | 20.560 | 19.665 | 20.264 | 19.844 | 20.530 |
| Total LY disc | 21.902 | 21.914 | 22.022 | 22.042 | 21.901 | 21.914 | 22.020 | 22.041 | 21.899 | 21.914 | 22.016 | 22.041 |
| **Costs (€)** |  |  |  |  |  |  |  |  |  |  |  |  |
| Drug and administration costs | 17126.87 | 17533.90 | 17126.86 | 17533.90 | 18257.86 | 18694.12 | 18257.86 | 18694.12 | 20093.32 | 20576.46 | 20093.32 | 20576.46 |
| Heart failure costs | 643.36 | 173.53 | 695.04 | 97.73 | 687.69 | 186.14 | 751.34 | 105.26 | 736.88 | 198.13 | 826.65 | 114.51 |
| Death costs | 4259.91 | 4251.36 | 4197.45 | 4183.66 | 4263.82 | 4254.57 | 4200.33 | 4185.25 | 4266.67 | 4256.72 | 4207.63 | 4190.46 |
| Total costs | 22030.14 | 21958.79 | 22019.36 | 21815.26 | 23209.37 | 23134.83 | 23209.53 | 22984.63 | 25096.86 | 25031.30 | 25127.60 | 24881.42 |
| **Incrementals** |  |  |  |  |  |  |  |  |  |  |  |  |
| QALYs | 0.532 |  | 0.594 |  | 0.571 |  | 0.637 |  | 0.598 |  | 0.686 |  |
| Costs (€) | -71.35 |  | -204.10 |  | -74.54 |  | -224.89 |  | -65.56 |  | -246.17 |  |
| ICER (€) | -133.99 |  | -343.53 |  | -130.50 |  | -352.8 |  | -109.46 |  | -358.89 |  |

ALVD, asymptomatic left ventricular dysfunction; CHF, congestive heart failure; ICER, incremental cost effectiveness ratio; QALY, quality-adjusted life years

**D: Base Case results for Spain**

|  | **All anthracycline doses** | | | | **Anthracycline >100 mg/m^2^** | | | | **Anthracycline >250 mg/m^2^** | | | |
| --- | --- | --- | --- | --- | --- | --- | --- | --- | --- | --- | --- | --- |
|  | **Sarcoma patients** | | **Haematological malignancy patients** | | **Sarcoma** | | **Haematological malignancy patients** | | **Sarcoma** | | **Haematological malignancy patients** | |
|  | **Usual treatment** | **Usual treatment + dexrazoxane** | **Usual treatment** | **Usual treatment + dexrazoxane** | **Usual treatment** | **Usual treatment + dexrazoxane** | **Usual treatment** | **Usual treatment + dexrazoxane** | **Usual treatment** | **Usual treatment + dexrazoxane** | **Usual treatment** | **Usual treatment + dexrazoxane** |

| **Clinical events** |  |  |  |  |  |  |  |  |  |  |  |  |
| --- | --- | --- | --- | --- | --- | --- | --- | --- | --- | --- | --- | --- |
| Proportion with CHF (%) | 21.90 | 6.08 | 24.38 | 3.27 | 23.54 | 6.57 | 26.10 | 3.57 | 24.83 | 6.97 | 28.17 | 3.81 |
| Average age at CHF diagnosis (years) | 57.44 | 59.91 | 56.57 | 58.67 | 57.41 | 59.97 | 56.48 | 59.03 | 57.13 | 59.91 | 55.99 | 58.90 |
| Years of life with CHF | 4.90 | 1.24 | 5.45 | 0.70 | 5.25 | 1.33 | 5.86 | 0.74 | 5.59 | 1.41 | 6.42 | 0.79 |
| Number of CHF hospitalizations | 0.61 | 0.19 | 0.67 | 0.12 | 0.65 | 0.21 | 0.73 | 0.13 | 0.69 | 0.22 | 0.80 | 0.14 |
| Average age at death (years) | 68.94 | 69.01 | 67.43 | 67.56 | 68.93 | 69.01 | 67.42 | 67.55 | 68.93 | 69.01 | 67.39 | 67.55 |
| **Cause of death (%)** |  |  |  |  |  |  |  |  |  |  |  |  |
| Death from cancer | 56.38 | 57.05 | 56.12 | 57.06 | 56.30 | 57.02 | 56.07 | 57.04 | 56.26 | 57.01 | 55.97 | 57.04 |
| Cardiac death | 5.42 | 4.17 | 5.69 | 3.92 | 5.57 | 4.20 | 5.80 | 3.94 | 5.69 | 4.22 | 6.00 | 3.95 |
| Death from infection or respiratory disease | 2.61 | 2.68 | 2.59 | 2.73 | 2.61 | 2.69 | 2.58 | 2.73 | 2.58 | 2.68 | 2.62 | 2.73 |
| Death from other disease | 28.07 | 28.49 | 28.11 | 28.67 | 28.00 | 28.49 | 28.06 | 28.68 | 27.95 | 28.49 | 27.93 | 28.67 |
| Violent death | 7.52 | 7.61 | 7.49 | 7.62 | 7.52 | 7.60 | 7.49 | 7.61 | 7.52 | 7.60 | 7.48 | 7.61 |
| Death from unknown cause | 0.00 | 0.00 | 0.00 | 0.00 | 0.00 | 0.00 | 0.00 | 0.00 | 0.00 | 0.00 | 0.00 | 0.00 |
| **QALYs** |  |  |  |  |  |  |  |  |  |  |  |  |
| QALYs without cardiac disease | 18.380 | 19.511 | 18.529 | 19.768 | 18.213 | 19.421 | 18.355 | 19.679 | 18.079 | 19.343 | 18.150 | 19.567 |
| QALYs with ALVD | 1.044 | 0.720 | 1.094 | 0.773 | 1.165 | 0.775 | 1.171 | 0.835 | 1.175 | 0.823 | 1.261 | 0.915 |
| QALYs with CHF | 0.361 | 0.087 | 0.368 | 0.044 | 0.388 | 0.092 | 0.396 | 0.046 | 0.411 | 0.097 | 0.434 | 0.049 |
| Total QALYs | 19.785 | 20.317 | 19.991 | 20.585 | 19.717 | 20.288 | 19.923 | 20.561 | 19.665 | 20.264 | 19.844 | 20.530 |
| Total LY disc | 21.903 | 21.915 | 22.022 | 22.043 | 21.901 | 21.915 | 22.021 | 22.042 | 21.900 | 21.914 | 22.017 | 22.042 |
| **Costs (€)** |  |  |  |  |  |  |  |  |  |  |  |  |
| Drug and administration costs | 10469.07 | 11313.61 | 10469.08 | 11313.62 | 11133.52 | 12038.71 | 11133.52 | 12038.71 | 12211.83 | 13214.29 | 12211.83 | 13214.29 |
| Heart failure costs | 1213.51 | 315.76 | 1276.84 | 169.37 | 1316.62 | 341.99 | 1377.64 | 183.86 | 1383.84 | 354.18 | 1511.58 | 198.13 |
| Death costs | 4259.52 | 4251.05 | 4197.10 | 4183.30 | 4263.43 | 4254.28 | 4199.98 | 4184.93 | 4266.23 | 4256.42 | 4207.25 | 4190.12 |
| Total costs | 15942.11 | 15880.43 | 15943.02 | 15666.29 | 16713.57 | 16634.98 | 16711.14 | 16407.51 | 17861.91 | 17824.89 | 17930.67 | 17602.55 |
| **Incrementals** |  |  |  |  |  |  |  |  |  |  |  |  |
| QALYs | 0.532 |  | 0.594 |  | 0.571 |  | 0.637 |  | 0.599 |  | 0.686 |  |
| Costs (€) | -61.68 |  | -276.73 |  | -78.59 |  | -303.63 |  | -37.02 |  | -328.12 |  |
| ICER (€) | -115.82 |  | -465.69 |  | -137.58 |  | -476.35 |  | -61.80 |  | -478.28 |  |

ALVD, asymptomatic left ventricular dysfunction; CHF, congestive heart failure; ICER, incremental cost effectiveness ratio; QALY, quality-adjusted life years

**E: Base Case results for the United Kingdom**

|  | **All anthracycline doses** | | | | **Anthracycline >100 mg/m^2^** | | | | **Anthracycline >250 mg/m^2^** | | | |
| --- | --- | --- | --- | --- | --- | --- | --- | --- | --- | --- | --- | --- |
|  | **Sarcoma patients** | | **Haematological malignancy patients** | | **Sarcoma** | | **Haematological malignancy patients** | | **Sarcoma** | | **Haematological malignancy patients** | |
|  | **Usual treatment** | **Usual treatment + dexrazoxane** | **Usual treatment** | **Usual treatment + dexrazoxane** | **Usual treatment** | **Usual treatment + dexrazoxane** | **Usual treatment** | **Usual treatment + dexrazoxane** | **Usual treatment** | **Usual treatment + dexrazoxane** | **Usual treatment** | **Usual treatment + dexrazoxane** |

| **Clinical events** |  |  |  |  |  |  |  |  |  |  |  |  |
| --- | --- | --- | --- | --- | --- | --- | --- | --- | --- | --- | --- | --- |
| Proportion with CHF (%) | 21.81 | 6.07 | 24.29 | 3.27 | 23.45 | 6.56 | 26.02 | 3.57 | 24.75 | 6.96 | 28.08 | 3.81 |
| Average age at CHF diagnosis (years) | 57.35 | 59.89 | 56.49 | 58.67 | 57.33 | 59.95 | 56.41 | 59.03 | 57.06 | 59.89 | 55.92 | 58.90 |
| Years of life with CHF | 4.87 | 1.23 | 5.42 | 0.69 | 5.22 | 1.33 | 5.82 | 0.74 | 5.56 | 1.40 | 6.38 | 0.79 |
| Number of CHF hospitalizations | 0.63 | 0.20 | 0.68 | 0.13 | 0.65 | 0.21 | 0.72 | 0.12 | 0.70 | 0.22 | 0.80 | 0.14 |
| Average age at death (years) | 68.81 | 68.88 | 67.31 | 67.44 | 68.80 | 68.88 | 67.30 | 67.44 | 68.79 | 68.87 | 67.28 | 67.43 |
| **Cause of death (%)** |  |  |  |  |  |  |  |  |  |  |  |  |
| Death from cancer | 56.39 | 57.06 | 56.13 | 57.07 | 56.31 | 57.03 | 56.08 | 57.05 | 56.27 | 57.02 | 55.98 | 57.05 |
| Cardiac death | 5.47 | 4.12 | 5.70 | 3.92 | 5.62 | 4.23 | 5.81 | 3.95 | 5.74 | 4.25 | 6.01 | 3.96 |
| Death from infection or respiratory disease | 2.57 | 2.67 | 2.58 | 2.73 | 2.57 | 2.67 | 2.57 | 2.72 | 2.54 | 2.66 | 2.61 | 2.72 |
| Death from other disease | 28.04 | 28.47 | 28.09 | 28.66 | 27.97 | 28.47 | 28.04 | 28.67 | 27.92 | 28.47 | 27.91 | 28.66 |
| Violent death | 7.53 | 7.61 | 7.50 | 7.62 | 7.53 | 7.60 | 7.50 | 7.61 | 7.53 | 7.60 | 7.49 | 7.61 |
| Death from unknown cause | 0.00 | 0.00 | 0.00 | 0.00 | 0.00 | 0.00 | 0..00 | 0.00 | 0.00 | 0.00 | 0.00 | 0.00 |
| **QALYs** |  |  |  |  |  |  |  |  |  |  |  |  |
| QALYs without cardiac disease | 18.373 | 19.500 | 18.523 | 19.759 | 18.206 | 19.410 | 18.350 | 19.671 | 18.072 | 19.334 | 18.145 | 19.558 |
| QALYs with ALVD | 1.041 | 0.718 | 1.091 | 0.771 | 1.114 | 0.773 | 1.169 | 0.833 | 1.172 | 0.821 | 1.259 | 0.913 |
| QALYs with CHF | 0.359 | 0.086 | 0.367 | 0.043 | 0.387 | 0.092 | 0.395 | 0.046 | 0.410 | 0.097 | 0.433 | 0.049 |
| Total QALYs | 19.774 | 20.305 | 19.982 | 20.574 | 19.707 | 20.276 | 19.915 | 20.550 | 19.655 | 20.252 | 19.836 | 20.520 |
| Total LY disc | 21.886 | 21.898 | 22.009 | 22.028 | 21.885 | 21.898 | 22.007 | 22.028 | 21.884 | 21.897 | 22.003 | 22.028 |
| **Costs (€)** |  |  |  |  |  |  |  |  |  |  |  |  |
| Drug and administration costs | 2767.52 | 4337.20 | 2767.52 | 4337.19 | 2841.53 | 4523.93 | 2841.53 | 4523.93 | 2961.65 | 4824.83 | 2961.65 | 4824.83 |
| Heart failure costs | 689.08 | 177.39 | 715.72 | 98.11 | 737.10 | 191.33 | 768.84 | 100.41 | 781.89 | 199.40 | 848.03 | 108.97 |
| Death costs | 3211.78 | 3205.77 | 3163.48 | 3153.35 | 3214.71 | 3208.19 | 3165.69 | 3154.56 | 3216.76 | 3209.81 | 3171.20 | 3158.46 |
| Total costs | 6668.38 | 7720.36 | 6646.73 | 7588.66 | 6793.35 | 7823.46 | 6776.06 | 7778.90 | 6960.30 | 8234.04 | 6977.88 | 8092.29 |
| **Incrementals** |  |  |  |  |  |  |  |  |  |  |  |  |
| QALYs | 0.530 |  | 0.592 |  | 0.570 |  | 0.635 |  | 0.596 |  | 0.683 |  |
| Costs (€) | 1051.98 |  | 941.93 |  | 1130.11 |  | 1002.84 |  | 1273.74 |  | 1114.37 |  |
| ICER (€) | 1983.26 |  | 1590.85 |  | 1986.40 |  | 1578.82 |  | 2135.24 |  | 1629.80 |  |

ALVD, asymptomatic left ventricular dysfunction; CHF, congestive heart failure; ICER, incremental cost effectiveness ratio; QALY, quality-adjusted life years
